# *NOTCH2NLC* GGC repeat expansion causes retinal pathology with intranuclear inclusions throughout the retina and causes visual impairment

**DOI:** 10.1186/s40478-023-01564-3

**Published:** 2023-05-02

**Authors:** Jun Sone, Shinji Ueno, Akio Akagi, Hiroaki Miyahara, Chisato Tamai, Yuichi Riku, Hiroyuki Yabata, Ryuichi Koizumi, Tomohiro Hattori, Hiroshi Hirose, Yoshito Koyanagi, Rei Kobayashi, Hisashi Okada, Yoshiyuki Kishimoto, Yoshio Hashizume, Gen Sobue, Mari Yoshida, Yasushi Iwasaki

**Affiliations:** 1 Department of Neuropathology, Institute for Medical Science of Aging, Aichi Medical University, 1-1 Yazakokarimata, Nagakute, Aichi 480-1195 Japan; 2 Department of Neurology, National Hospital Organization Nagoya Medical Center, 4-1-1, Sannomaru, Naka-Ku, Nagoya, Aichi 460-0001 Japan; 3 Department of Neurology, National Hospital Organization Suzuka National Hospital, 3-2-1, Kasado, Suzuka, Mie 513-8501 Japan; 4 Department of Neurology, Nagoya University Graduate School of Medicine, 65 Tsurumai, Showa-Ku, Nagoya, Aichi 466-8560 Japan; 5 Department of Ophthalmology, Nagoya University Graduate School of Medicine, 65 Tsurumai, Showa-Ku, Nagoya, Aichi 466-8560 Japan; 6 Department of Ophthalmology, Hirosaki University Graduate School of Medicine, 5 Zaifu, Hirosaki, Aomori 036-8562 Japan; 7 Department of Neurology, Shiga University of Medical Science. Seta-Tsukinowa, Otsu, 520-2192 Japan; 8 Department of Neurology and Stroke Medicine, Yokohama City University Graduate School of Medicine, 22-2 Seto, Kanazawa-Ku, Yokohama, Kanagawa 236-0027 Japan; 9 Department of Ophthalmology, National Hospital Organization Nagoya Medical Center, 4-1-1, Sannomaru, Naka-Ku, Nagoya, Aichi 460-0001 Japan; 10 Department of Neuropathology, Choju Medical Institute, Fukushimura Hospital, 19-14, Yamanaka, Noyori, Toyohashi, Aichi 441-8124 Japan; 11 Aichi Medical University, 1-1 Yazakokarimata, Nagakute, Aichi 480-1195 Japan

**Keywords:** *NOTCH2NLC*, GGC repeat, Neuronal intranuclear inclusion disease, Retinitis Pigmentosa, Leukoencephalopathy, Diffusion weighted image, Retinal pathology, p-62, Gliosis

## Abstract

The retinal pathology of genetically confirmed neuronal intranuclear inclusion disease (NIID) is yet unknown. We report the ocular findings in four NIID patients with *NOTCH2NLC* GGC repeat expansion to investigate the pathology of retinopathy. All four NIID patients were diagnosed by skin biopsy and *NOTCH2NLC* GGC repeat analysis. Ocular findings in patients with NIID were studied using fundus photographs, optical coherence tomographic images (OCT), and full-field electroretinograms (ERGs). The histopathology of the retina was studied on autopsy samples from two cases with immunohistochemistry. All patients had an expansion of the GGC repeat (87–134 repeats) in the *NOTCH2NLC.* Two patients were legally blind and had been diagnosed with retinitis pigmentosa prior to the diagnosis of NIID and assessed with whole exome sequencing to rule out comorbidity with other retinal diseases. Fundus photographs around the posterior pole showed chorioretinal atrophy in the peripapillary regions. OCT showed thinning of the retina. ERGs showed various abnormalities in cases. The histopathology of autopsy samples showed diffusely scattered intranuclear inclusions throughout the retina from the retinal pigment epithelium to the ganglion cell layer, and optic nerve glial cells. And severe gliosis was observed in retina and optic nerve. The *NOTCH2NLC* GGC repeat expansion causes numerous intranuclear inclusions in the retina and optic nerve cells and gliosis. Visual dysfunction could be the first sign of NIID. We should consider NIID as one of the causes of retinal dystrophy and investigate the GGC repeat expansion in *NOTCH2NLC*.

**Supplementary Information:**

The online version contains supplementary material available at 10.1186/s40478-023-01564-3.

## Introduction

Neuronal intranuclear inclusion disease (NIID) is a progressive neurodegenerative disease that is characterized by eosinophilic hyaline intranuclear inclusions in neuronal and somatic cells [17, 19, 22, 33, 34, 36]. Intranuclear inclusions were suggested to be composed of abnormal proteins including polyglycine protein in the nuclei and supposed to be related to the damage to the cells [4, 19]. A wide range of clinical manifestations of NIID, such as dementia, ataxia, developmental delay, parkinsonism, tremor, neuropathy, and autonomic dysfunction have been reported [33, 36], and they are said to be mainly associated with dementia that is observed in both sporadic and familial cases [2, 23, 33].

Skin biopsy has been used as a safe method to diagnose NIID [30, 33]. In addition, a characteristic, high-intensity signal in the corticomedullary junction on diffusion-weighted images (DWI) from brain MRI has become another helpful sign to diagnose NIID [30, 33]. In 2019, expanded GGC repeats in the *NOTCH2NLC* gene were reported as the cause of both the sporadic and familial NIID [12, 31, 32]. Currently, NIID can be diagnosed by genetic testing.

Retinal damage in NIID patients has been reported [9, 24]. Several studies reported a wide range of retinal abnormalities; some patients showed severe retinal dystrophy like retinitis pigmentosa (RP) while others were asymptomatic but had retinal changes [9, 18, 39, 40]. These studies have reported abnormal ERGs in NIID patients, indicating dysfunction of cells in the inner nuclear layer (INL) [3, 8, 10, 18, 39]. Only one NIID case had reported about histopathological findings of retina, but that case was genetically unconfirmed and child case [9]. And the underlying mechanisms of NIID retinopathy have not been clarified. To elucidate these observations of NIID, retinal histopathological studies of genetically confirmed NIID cases were needed.

In this study, we examined the retinal findings in four NIID patients diagnosed by skin biopsy and genetic analysis of the *NOTCH2NLC* gene and further analysed the retinal histology of two cases by autopsy.

## Methods

### Patients

We include four sporadic adult-onset NIID cases. All patients were assessed by skin biopsy and genetic testing of the *NOTCH2NLC* GGC repeats, both with positive results. In two of these cases, consent was obtained for eye examination at autopsy, and in the other two cases, RP was diagnosed prior to the diagnosis of NIID. They were recruited from National Hospital Organization Nagoya Medical Centre, Nagoya University Hospital, and Fukushimura Hospital, from 2013 to 2021with written informed consent of all patients. The protocol of this study was approved by the institutional review board of each institute. Clinical manifestations of Case 1 were partially reported [2].

All patients underwent ophthalmic screenings. The medical histories and the results of the clinical examinations including neurological findings, brain MRI, the best-corrected visual acuity (BCVA), slit-lamp examinations, ophthalmoscopy, ERGs, spectral domain optical coherence tomographic (SD-OCT) images, and fundus photographs were analysed. Full-field ERGs were recorded from patients who conformed to the guidelines of the International Society for Clinical Electrophysiology of Vision Standards [20]. All patients underwent colour fundus photography and SD-OCT imaging (Spectralis, Heidelberg Engineering, Heidelberg, Germany or Cirrus HD-OCT; Carl Zeiss Meditec, Dublin, CA). Case 3 had fundus autofluorescence (FAF) imaging performed (Optos 200Tx, Optos PLC, Dunfermline, UK).

### Histological analysis

Skin biopsy was performed and assessed in the same manner as in a previous report [34]. Cases 1 and 2 were examined by autopsy with post-mortem intervals < 12 h. Histopathological examination including immunohistochemistry with anti-p62 antibody, anti-ubiquitin antibody, anti-GFAP (glial fibrillary acidic protein) antibody and electron microscopic examination were performed in the same manner as in previous reports [19, 29, 33, 34]. Eyeballs were fixed in 10% neutral buffered formalin and were opened in the horizontal plane, embedded in paraffin and sectioned in 6-μm thickness. The microscopic sections through the pupil-optic nerve-macula plane was prepared. The retina was partially detached from the retinal pigment epithelium (RPE) due to postmortem artifacts.

For the assessment of intranuclear inclusion frequency, we selected 12 random microscopic fields of the retina. For the assessment of intranuclear inclusion frequency in the glial cells in the optic nerve and brain frontal lobe, 10 random microscopic fields were used and analysed. The numbers of the nucleus of the retinal ganglion cell layer (GCL), inner nuclear layer (INL), outer nuclear layer (ONL), and optic nerve head, neurons and astrocytes in frontal lobe, and the number of cells with p62-positive intranuclear inclusion were counted. The ratio of the number of cells with p62-positive intranuclear inclusion per the number of cells was calculated for each image.

### Genetic analyses

Genomic DNA of peripheral blood leukocytes was extracted from NIID patients, and GGC repeat expansion in *NOTCH2NLC* was assessed by repeat primed PCR, and the number of GGC repeat expansions was confirmed with fluorescence amplicon length analysis as described in detail in a previous report [31]. In Cases 3 and 4, whole exome sequencing (WES) by NovaSeq 6000 and Agilent Sure Select Human All Exon V6 + UTR was performed, and data were analysed using 329 retinal disease-causing genes registered in Retinal Information Network (RetNet database [27]) to screen the pathogenic variants that can cause the retinal degeneration. We performed a visual inspection of pathogenic variant sites using the Integrative Genomics Viewer (IGV). The methods have been described in detail [15]. About ATXN7, one of the RetNet gene and the cause of spinocerebellar ataxia type 7 (SCA7), we investigated CAG repeat sequence expansion using a reported PCR method [14].

## Results

**Fig. 1:**
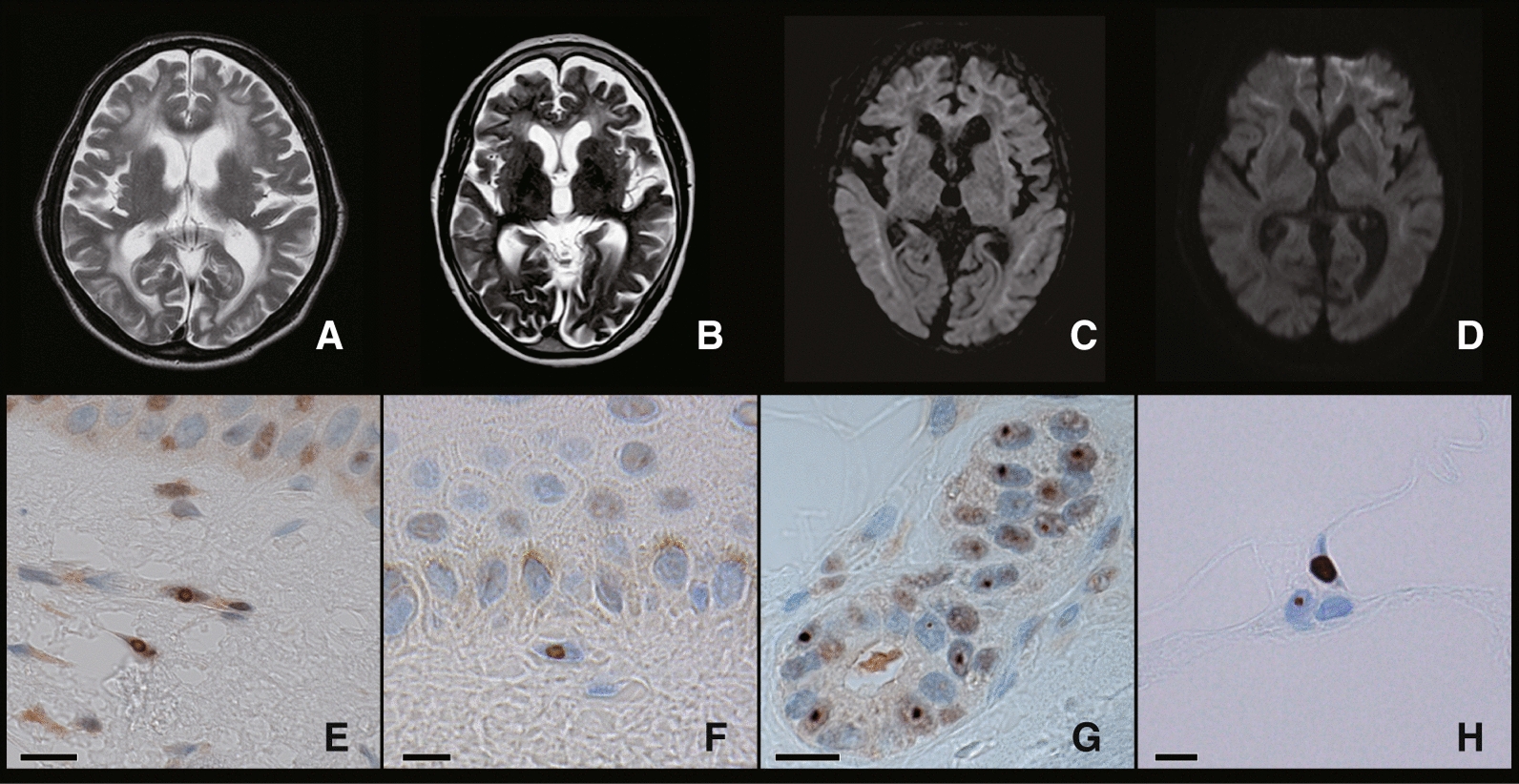
NIID clinical findings. The findings of brain MRI and skin biopsy. A-B: T2 weighted images showed leukoencephalopathy (**A** Case 1 and **B** Case 4). **C**–**D** D*iffusion-weighted imaging* (DWI) showed an abnormally high-intensity signal in the corticomedullary junction (**C** Case 2 and **D** Case 3). **E**–**H** Skin biopsy findings of NIID cases. Immunostaining showed intranuclear inclusions in the fibroblasts (**E**: Case 2, **F**: Case 3) with anti-ubiquitin antibody, sweat gland cells (**G**: Case 1), and adipocytes (**H**: Case 4) with anti-p-62 antibody. Scale bars: 10 μm

The summary of the demographic and clinical characteristics of the patients is shown in Table 1. All patients showed leukoencephalopathy in the T2 image of brain MRI and characteristic abnormal high-intensity signal in the corticomedullary junction in DWI (Fig. 1A–D), and these MRI findings led to the diagnosis of NIID. Skin biopsy samples showed intranuclear inclusions confirmed by anti-p62 staining (Fig. 1E–H), and they had heterozygous GGC repeat expansion in the *NOTCH2NLC* gene (Table 1 and Fig. 1I). The number of expanded GGC repeat sequence in these NIID cases were from 87 to 134 (Table 1). Cases 3 and 4 had been diagnosed earlier with RP from their ocular findings prior to the diagnosis of NIID (Table 2). All four patients did not have ATXN7 CAG repeat expansions [21].

**Table 1:** Summary of NIID cases

Patient	Sex	Age at diagnosis of NIID (Years)	Age at ophthalmology exam (Years)	*NOTCH2NLC* GGC repeat number		Neurological findings
				Non-expanded	Expanded	
Case 1	M	67	81	13	97	Cognitive dysfunction, Ataxia, Aphasia, Bladder dysfunction
Case 2	F	75	78	13	87	Cognitive dysfunction, Ataxia, Bladder dysfunction
Case 3	F	67	72	9	134	Cognitive dysfunction, Ataxia
Case 4	F	75	75	7	119	Cognitive dysfunction, Ataxia

**Table 2:** Summary of ophthalmologic findings

Patient	Sex	Age at visual symptom (Years)	BVCA (Decimal)		Fundus image	OCT findings	Average retinal thickness (μm)	ERG	Other ocular disease
			Right	Left					
Case 1	M	–	1	0.8	No specific abnormality	Thinning of retinal thickness	209	Severely reduced in both rod and cone function	–
Case 2	F	78	0.2	0.3	No specific abnormality	Thinning of retinal thickness	226.9	n.a	Cataract
Case 3	F	52	0.03	0.2	Sever retinal degeneration with macular atrophy	Sever ellipsoid zone loss and thinning of outer nuclear layer	n.a	Extinguished	Intraocular lens
Case 4	F	60	0.05	0.02	Sever retinal degeneration with macular atrophy	Sever ellipsoid zone loss and thinning of total retinal thickness	179	Severely reduced in both rod and cone function	Intraocular lens

n.a.: not available

### Presentation of each NIID cases

**Fig. 2:**
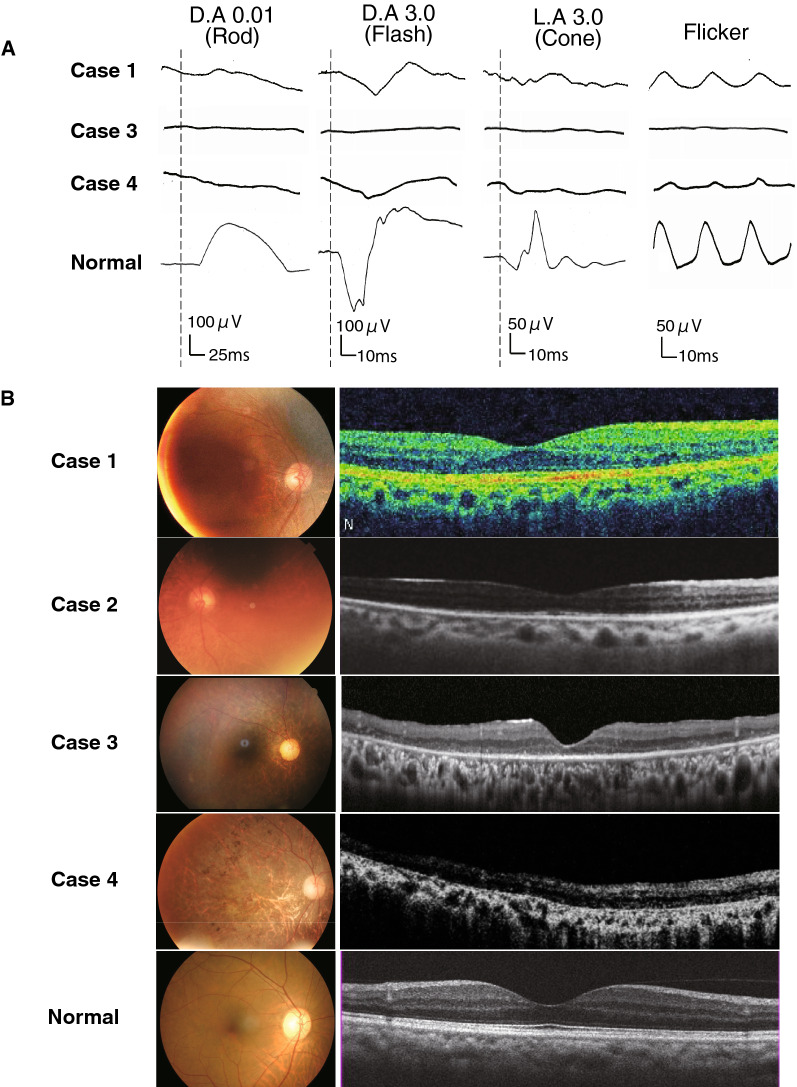
NIID ophthalmic findings. **A**; Full-field ERGs recorded from NIID Cases 1, 3, and 4, and 1 normal control. Case 3 with severe retinal dystrophy had non-recordable ERGs and severely reduced ERGs (Case 4) for all stimulus conditions. ERGs of Case 1 were reduced for all the stimulus conditions. **B**; Fundus images and OCT images of NIID cases. Fundus photographs from one eye with the better-quality image and horizontal OCT images are shown. Chorioretinal atrophies in the peripapillary regions are seen in the fundus images of Case 1, otherwise, fundus images show no abnormalities. Fundus images of Case 3 showed severe retinal degeneration. A fundus image of Case 4 showed severe macular atrophy. OCT showed partial disruption of the ellipsoid zone (EZ) in Case 1, and absence of the EZ and thinning of the outer nuclear layer in Cases 3 and 4

Case 1 had neither a family history of dementia nor a past history of retinal disease. He presented dementia from the age of 67 [2]. At the age of 71, he presented with gait disturbance. At the age of 74, he presented bilateral miosis. Manual muscle testing was normal, although hyporeflexia, hypopallesthesia, wide-based gait, limb ataxia, and dysdiadochokinesia were observed. He was diagnosed with neurogenic bladder and required urinary catheterization [2]. Brain MRI showed leukoencephalopathy (Fig. 1A) and abnormal hyperintensity in the corticomedullary junction in DWI. A nerve conduction study revealed reduced motor conduction velocities. Skin biopsy showed intranuclear inclusions (Fig. 1G), and *NOTCH2NLC* GGC repeat expansion was observed. Afterward, aphasia and explosive speech gradually became apparent, the decline in word recall became noticeable, and verbal communication gradually became difficult. The scores for Mini-Mental State Examination (MMSE) and Frontal Assessment Battery (FAB) gradually deteriorated (at the age of 77, MMSE 8/30, and FAB 0/18). He had repeated vomiting attacks and required multiple hospitalizations. At the age of 79, an ophthalmology examination showed that the decimal BCVAs were relatively well preserved at above 0.6 in 2 eyes (Table 2). The ERGs were noisy due to cognitive dysfunction. Dark-adapted (D.A) 0.01 ERG (rod) were reduced, and the a- and b-waves of the D.A 10.0 ERG were reduced. And the amplitudes of the b-wave of the light-adapted (L.A)3.0 ERG and flicker ERG were moderately reduced. These ERGs recorded showed reductions for all stimulus conditions which suggested widespread photoreceptor degeneration (Fig. 2A). Fundus image showed peripapillary chorioretinal atrophy and that was less than 1.0-disc diameter. OCT image showed reduced retinal thickness and average thickness was 209 μm (Fig. 2B and Table 2).

Case 1 passed away from dehydration at the age of 81 and was studied by an autopsy.

Case 2 had no family history of neurological disease. At the age of 75, she presented encephalitic episode with headache, high fever, staggering gait, and loss of appetite, and was hospitalized for two days. She presented disorientation and miosis. At the age of 76, she was unable to manage her medications on her own. The score of MMSE was 14/30, and brain MRI findings showed leukoencephalopathy and high intensity in the corticomedullary junction in DWI, which were consistent with NIID (Fig. 1C). She was diagnosed with NIID based on the MRI findings, positive skin biopsy results (Fig. 1E), and later, *NOTCH2NLC* GGC repeat expansion. Two months later, she presented an encephalitic episode again and presented abnormal behaviour such as sleeping naked, inconsistent speech, and dizziness for three days, which were resolved spontaneously. At the age of 78, she had difficulty urinating and was diagnosed with a neurogenic bladder. At the age of 79, she consulted an ophthalmologist for cataract treatment, who noted her miosis and thinning of the retina, and normal findings of the fundus image (Fig. 2), and did not undergo ERG. OCT image showed reduced retinal thickness and average thickness was 226.9 μm (Fig. 2B and Table 2). A month later, she was hospitalized again with a loss of appetite and worsening gait. She presented wide-based and petite gait, hyporeflexia, and bilateral extensor plantar reflex. She sometimes entered someone else's room by mistake, was unable to dress, and made incoherent remarks. After her hospitalization, she had onsets and remissions of encephalitic episodes on at least 5 occasions over a 3-year period. She died of poor oral intake and malnutrition at the age of 82 and underwent an autopsy.

Case 3, who had noticed night blindness and visual field constriction, was diagnosed with RP at the age of 52, 15 years before the diagnosis of NIID. She had no family history of retinal dystrophy. She complained of a progressive constriction of the visual field and reduction of visual acuity after diagnosis of RP. At the age of 67, she was suspected of having NIID from the characteristic, abnormal high-intensity signal in the DWI of brain MRI (Fig. 1D) during a screening of paroxysmal positional vertigo. She was diagnosed with NIID by skin biopsy findings (Fig. 1F), and later, by *NOTCH2NLC* GGC repeat expansion. At the age of 72, her vision fell to hand motion visual acuity, and ERGs elicited with any stimulus condition were extinguished (less than 10 μV) and it was difficult to determine which photoreceptor, rod or cone, was more affected (Fig. 2A). Fundus images showed retinal degeneration with macular atrophy along with chorioretinal atrophy in the peripapillary regions. OCT showed the absence of the ellipsoid zone in the cones, and a severe reduction of the outer nuclear layer thickness in the macular area which corresponded to the fundus images of the macular atrophy.

Case 4 noticed a reduction of her visual acuity and an impairment of the central visual fields at the age of 60 without a family history of retinal dystrophy. She was diagnosed with RP with macula atrophy, and her visual acuity gradually decreased thereafter. She was reported to have dementia at around the age of 70 and was diagnosed with NIID based on the brain MRI image (Figs. 1B) and the skin biopsy findings (Figs. 1H) at the age of 75, and *NOTCH2NLC* GGC repeat expansion. The ERGs of Case 4 which showed DA 0.01 ERGs were non-recordable, and the ERGs elicited by DA3.0, LA3.0, and 30 Hz flicker were severely reduced (Fig. 2A). The remaining photopic ERG indicated rod-cone degeneration, which was inconsistent with the fundus image showing severe retinal degeneration observed mainly in the macula. Fundus images showed retinal degeneration with macular atrophy along with peripapillary chorioretinal atrophy. OCT showed almost same findings of Case 3, the absence of the ellipsoid zone, and a severe reduction of the outer nuclear layer thickness in the macular area, and average thickness was 179 μm (Fig. 2B and Table 2).

WES with targeted analysis was performed on Cases 3 and 4. The analysis did not identify causative variants in the 329 genes causing retinal diseases (RetNet database [27]) in these two patients. All variants with a minor allele frequency ≤ 10% in gnomAD Total database [13] observed in this analysis are shown in Supplemental Table 1. All of these variants interpreted as benign in ClinVar [16], and the frequency of then in jMorp 38KJPN [35] were over 10%. No low-frequency variants for recessive genes (< 0.5%), and no low-frequency variants for dominant genes (< 0.01%) were detected in both of the cases.

### The autopsy findings

Macroscopic findings of the central nervous system showed frontal dominant cerebral atrophy, and marked atrophy of white matter and ventricular dilatation were observed in both Cases 1 and 2.

**Fig. 3:**
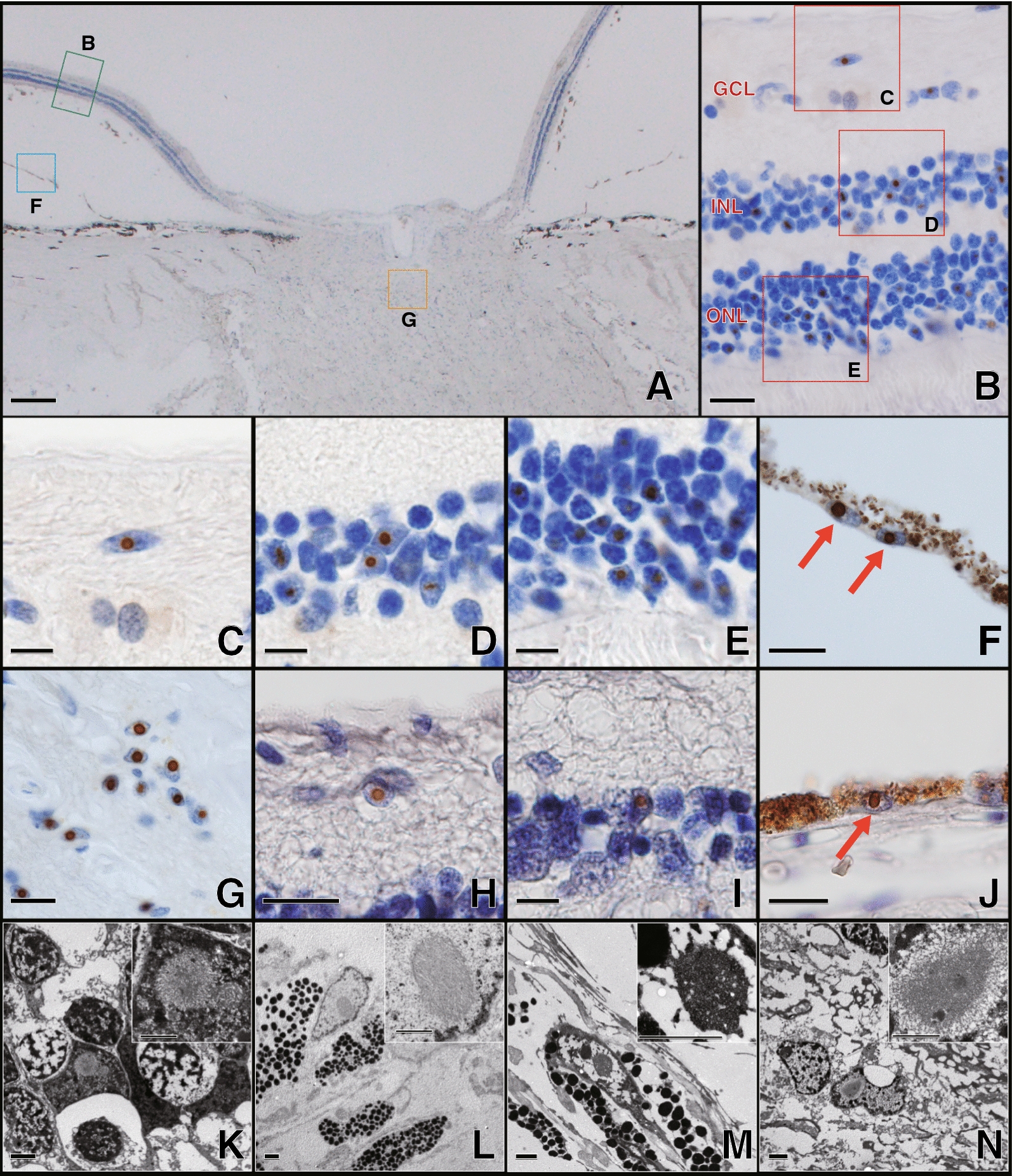
NIID Histopathological findings of ophthalmic organs. Case 1 (**A**–**G**, **K**–**L**, and **N**) and Case 2 (**H**–**J** and **M**) findings. **A**–**J**: Immunohistopathological study with anti-p62 antibody. **A** The horizontal section of the optic nerve. The right side is temporal. The area surrounded by the green square labeled **B** is magnified in Fig. 2B, the area surrounded by the blue square labeled **F** is magnified in Fig. 2F, and the area surrounded by the yellow square labeled **G** is magnified in Fig. 2G. **B**–**E**: **B**: Low magnification finding of the retina. The areas of the red squares labeled as **C**, **D**, and **E** are magnified in Figs. 2C, D, 2E respectively. **C** and **H**: Retinal ganglion cell layer (GCL). **D** and **I**: Inner nuclear layer (INL). **E**: Outer nuclear layer (ONL). **F** and **J**: Retinal pigment epithelium cells. Red arrow showed intranuclear inclusions of pigment epithelial cells. **G**: Optic nerve. **K**–**N**: Electric microscope findings. A higher magnification of the intranuclear inclusion is shown in the upper right corner of each figure. **K**: Inner nuclear layer. **L**–**M**: Retinal pigment epithelium cell. **N**: Glial cell in the optic nerve. p-62 positive intranuclear inclusions were observed in the retinal ganglion cells, cells in the inner nuclear layer and outer nuclear layer, and photoreceptor cells in the retina, and retinal pigment epithelium cells and glial cells in the optic nerve. These intranuclear inclusions were composed of fine filaments and without limiting membrane. Scale bars: **A**: 200 μm, **B**: 10 μm, **C**–**E**: 5 μm, **F**–**J**:10 μm, and **K**–**N**: 2.0 μm

In the retinal section, p-62 positive intranuclear inclusions were observed in the nuclei of the retinal ganglion cell layer (Fig. 3C: Case 1 and 3H: Case 2), outer nuclear layer (Fig. 3D: Case 1 and 3I: Case 2), inner nuclear layer (Fig. 3E: Case 1), and retinal pigment epithelium cells (Fig. 3F: Case 1 and 3J: Case 2) in both Case 1 and Case 2. In the optic nerve head, many inclusions were observed in the nucleus of the glial cells (Fig. 3G: Case 1). The electron microscopic findings showed that intranuclear inclusions consist of fine filaments without limiting membrane in the retina (Fig. 3K: Case 1), retinal pigment epithelium cells (Fig. 3L: Case 1 and 3M: Case 2), and glial cells of the optic nerve head (Fig. 3N: Case 1). These intranuclear inclusions appeared more frequently in Case 1. The frequency of intranuclear inclusion-positive cells in the outer nuclear layer was 25.4 ± 9.0% (Case 1) and 2.1 ± 1.5% (Case 2), that in the inner nuclear layer was 12.4 ± 5.0% (Case 1) and 3.7 ± 1.1% (Case 2), that in the retinal ganglion cell layer was 9.5 ± 9.0% (Case 1) and 10.5 ± 9.8% (Case 2), and optic nerve was 16.4 ± 9.0% (Case 1).

**Fig. 4:**
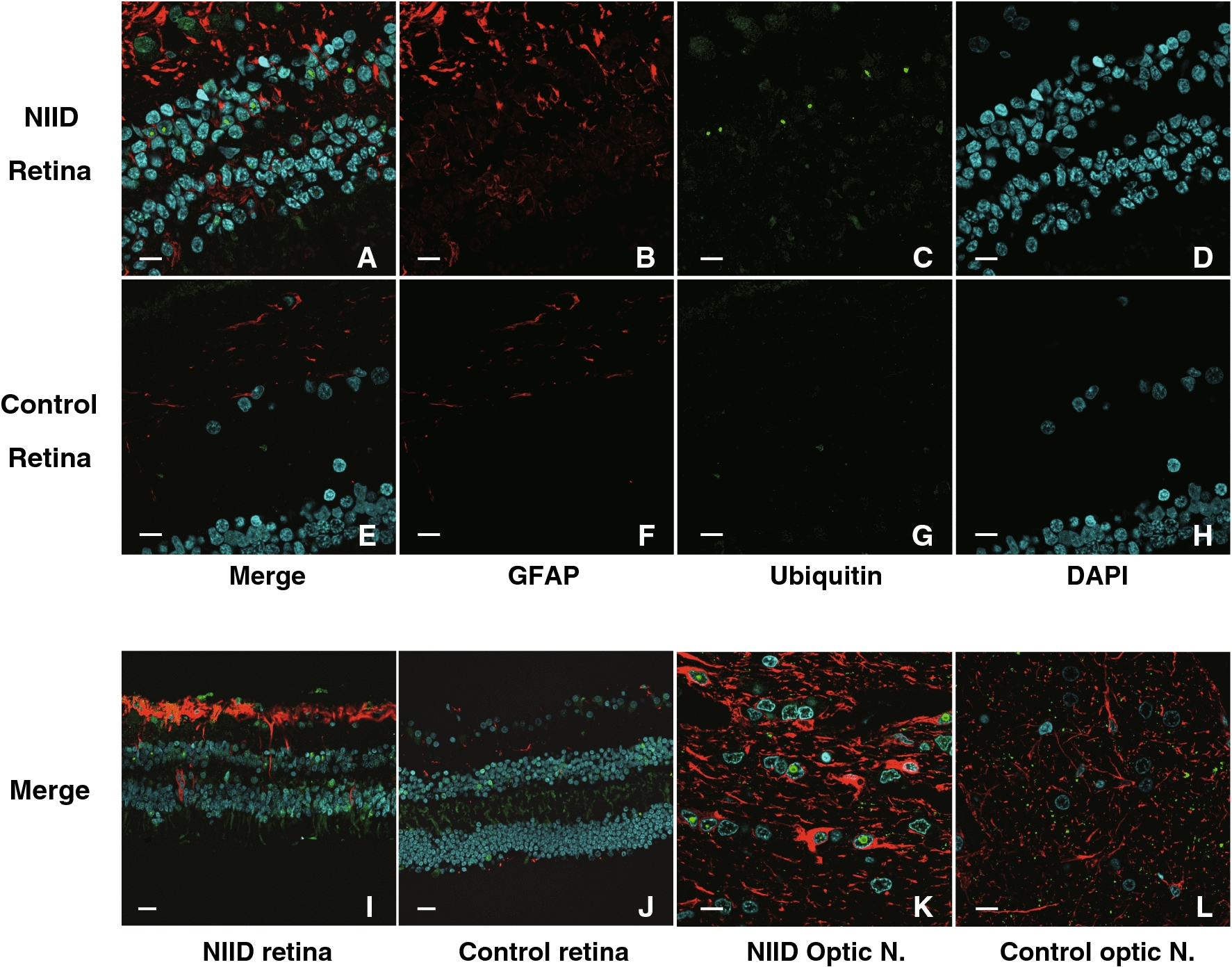
Immunohistopathological study with anti-GFAP antibody. Immunofluorescence study of retina and optic nerve with anti-GFAP antibody (red) and anti-ubiquitin antibody (green). **A** Merged view of Immunofluorescence stain of NIID retina (Case 1) with GFAP (**B**, red), with ubiquitin (**C**, green) and with DAPI (**D**, blue). Severe gliosis and ubiquitin-positive intranuclear inclusions were observed. **E**–**H**: The same Immunofluorescence stain of control retina. **I**–**J** Low magnification of NIID retina (**I**: Case 2) and control retina (**J**). **K**–**L**: Optic nerve of NIID (**K**: Case1) and control (**L**). Severe gliosis was observed in both retina and optic nerve of NIID cases. Scale bars: **A**–**H** and **K**–**L**: 10 μm, **I**–**J**: 20 μm

We also studied about the gliosis of retina and optic nerve (Fig. 4). In both NIID cases, severe gliosis was observed in retina and optic nerve compared to the control case in anti-GFAP immunohistochemistry. Especially, the gliosis in ganglionic layer of retina was conspicuous (Fig. 4A, I). Loss of ganglionic cell was not apparent in both cases.

**Fig. 5:**
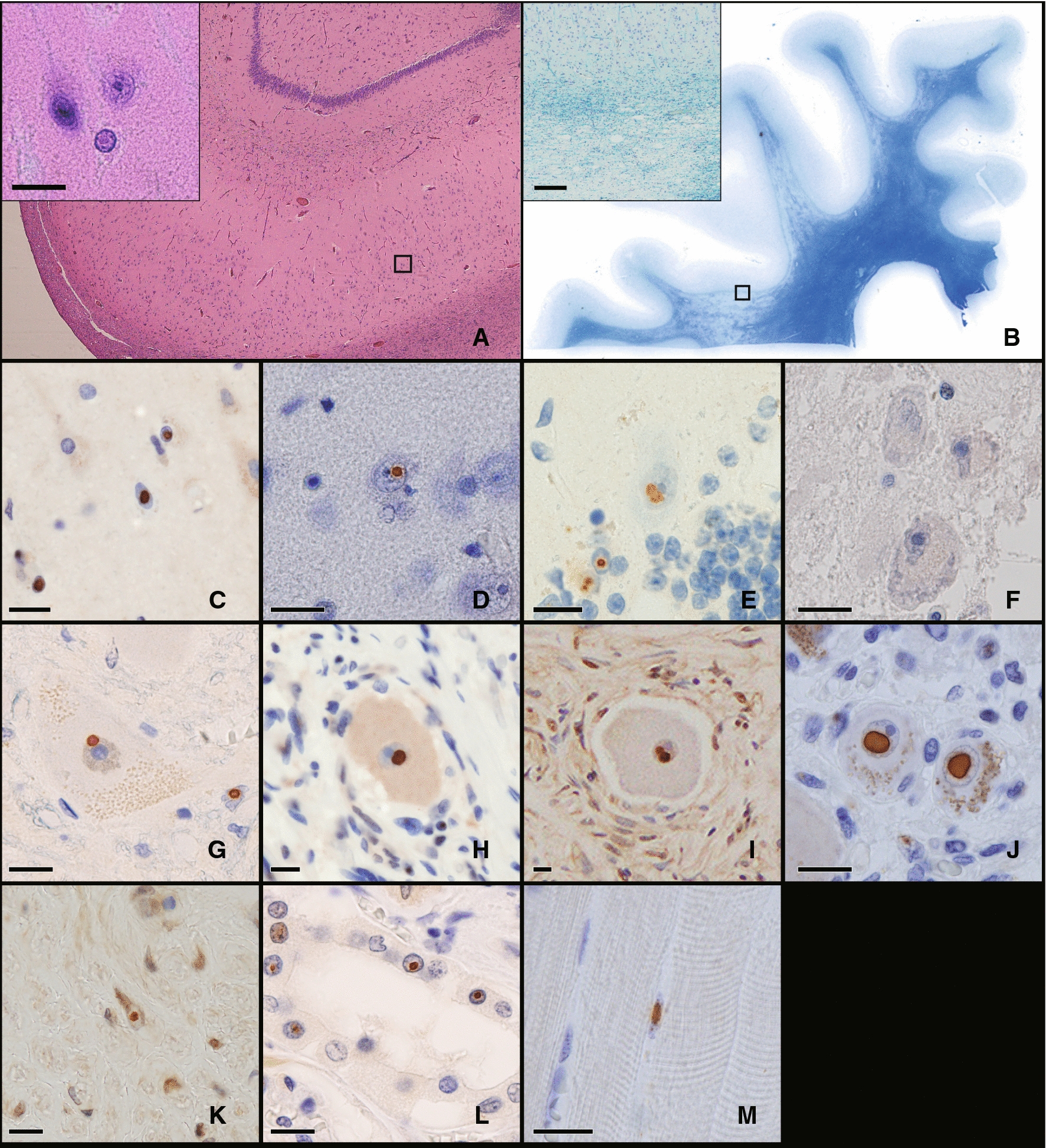
Autopsy findings. Autopsy findings of Case 1 and Case 2. The parts surrounded by the black squares in **A** and **B** are enlarged and shown in the upper left corner. **A** H&E stain of hippocampal CA1 of Case 1. The volume of the hippocampus was preserved. Eosinophilic intranuclear inclusions were observed chiefly in astrocytes. **B** Klüver-Barrera stain of Case 1 frontal lobe. Marked degeneration in the white matter was observed. **C**–**H**, **J**, and **L**–**M** Immunohistochemical staining with anti-p-62 antibody. **I** and **K** immunohistochemical staining with anti-ubiquitin antibody. **C** Neuron and astrocyte in the frontal lobe (Case 1). **D** Neuron in the frontal lobe (Case 2). **E** Purkinje cell and Bergmann glial cells (Case 1). **F** Neuron in Edinger–Westphal nucleus (Case 1). **G** Neuron in the spinal cord anterior horn (Case 1). **H** Dorsal root ganglion neuron (Case 1). **I** Dorsal root ganglion neuron (Case 2). **J** Sympathetic ganglion neurons (Case 1). **K** Schwann cells in the dorsal root of the spinal cord (Case 2). **L** Renal tubule cells in the kidney (Case 1). **M** Skeletal muscle cells in the iliopsoas (Case 1). p-62 positive or ubiquitin-positive intranuclear inclusion was observed in the neuron and astrocyte in the frontal lobe, Purkinje cells, spinal motor neurons, dorsal root ganglion (DRG), sympathetic ganglion neurons, Schwann cells, renal tubule cells, and skeletal muscle cells, but not in the neuron in the Edinger–Westphal nucleus. Scale Bars, **A** and **C**–**M**: 10 μm; **B**: 200 μm

Histopathological findings of the central nervous system showed that the volume and structure of hippocampus CA1 remained unchanged (Fig. 5A), and we diagnosed the cases with no Alzheimer's pathology because neurofibrillary tangles were observed only in the transentorhinal cortex, and senile plaques were not observed in both Case 1 and Case 2. Argyrophilic grain, Lewy body, and phosphorylated TDP43-positive constructs were not observed either. Myelin sheath degeneration and spongiform change of the cerebral white matter were observed (Fig. 5B) in both cases. Eosinophilic, ubiquitin-positive, and p-62-positive intranuclear inclusions were observed in the neurons and glial cells in the cerebral cortex (Fig. 5A, C, D), Purkinje cells (Fig. 5E), and motor neurons in the spinal cord (Fig. 5G). In the cerebellum, a decrease in Purkinje cells and proliferation of Bergmann glia were observed in both cases (Fig. 5E). However, in the Edinger-Westphal nucleus, no intranuclear inclusion was observed in the neurons and glial cells and seemed as intact (Fig. 5F) in Case 1. In Case 2, there were only a few intranuclear inclusions in the neurons. The frequency of intranuclear inclusion-positive neurons in the frontal cortex was 6.67 ± 3.6% (Case 1) and 2.85 ± 1.7% (Case 2), and astrocytes in the frontal cortex was 17.7 ± 5.7% (Case 1) and 10.1 ± 4.4% (Case 2),

Intranuclear inclusions were also observed in the peripheral nervous system such as in the dorsal root ganglia (Fig. 5H, I), sympathetic ganglion neurons (Fig. 5J) and Schwann cells (Fig. 5K), and cells in other organs such as the kidney (Fig. 5L) and skeletal muscles (Fig. 5M).

## Discussion

All NIID cases in this study were diagnosed by both skin biopsy findings and *NOTCH2NLC* GGC repeat expansion. Autopsy findings of Cases 1 and 2 showed widely distributed p-62 and ubiquitin-positive intranuclear inclusions. These findings were identical to the past NIID autopsy reports [17, 19, 33, 36], and the diagnosis of NIID was confirmed.

In retina, intranuclear inclusions were observed in the entire retinal layers, from the retinal pigment epithelium to the retinal ganglion cell layer in both two autopsied cases. In addition, an observation with electron microscopy of those intranuclear inclusions in the retina revealed the same findings as the intranuclear inclusions of NIID in previous reports [17, 22, 34]. These results indicated that *NOTCH2NLC* GGC expansion surely caused histopathological change and damage in the retina as well as in other nervous systems.

Only one young NIID case had been reported about the retinal histopathology that case presented dysarthria and nystagmus at the onset, age of 11 years, and examined at autopsy at the age of 21 years [9]. About this case, genetical tests including the GGC repeat expansion in *NOTCH2NLC* were not investigated. The intranuclear inclusions were found only in nervous system organs. About retina, intranuclear inclusions were found only in ganglion cells, but not in bipolar cells or retinal pigment cells in retina. Loss of ganglion cells, loss of retinal nerve fibres, and reactive astrocytosis were also reported.

About our cases in this study, intranuclear inclusions were found in nervous systems and also other organs. And in retina, intranuclear inclusions were more widely observed than previous reported case, that is, not only in ganglion cells, but in inner and outer cell layers including bipolar cells and in retinal pigment epithelium cells. Differences were observed in cell types in which intranuclear inclusions were observed between our case and previous case. Some neurodegenerative diseases such as fragile X-associated tremor/ataxia syndrome (FXTAS) and oculopharyngodistal myopathy (OPDM), were reported to present similar histopathological findings of NIID [5, 7, 28] showing p-62-positive intranuclear inclusions widely in nervous system and general organs. These diseases were reported to be caused by the expansion of the GGC repeat sequence on genes other than *NOTCH2NLC,* such as *FMR1, LRP12, GIPC1* and so on. Differences in pathological findings between previous young case and our cases may be due to the differences in genetic backgrounds between *NOTCH2NLC* and other genes, and such histopathological difference may be useful for differential diagnosis of these diseases in future. Further investigation in a larger number of cases is required.

Marked gliosis was observed in retina and optic nerve similar to previous report. These findings suggests that gliosis was induced as a result of tissue damage to the retina and optic nerve, and these histological changes may contribute to the ocular manifestations of NIID with *NOTCH2NLC* GGC repeat expansion [6].

The thinning of the outer nuclear layer in OCT in Case 1 seemed to be related to the accumulation of intranuclear inclusions in the outer nuclear layer cells, resulting in photoreceptor cell death, and may have affected the b-wave attenuation in the D.A 0.01. The b-wave in D.A 0.01 was known to be originated from the electrical activity of retinal outer nuclear layer bipolar cells, and the dysfunction of outer nuclear layer bipolar cells may have contributed to the reduced amplitude of b-wave in D.A 0.01 with or without impairment of the rod cells. However, the foveal microstructure of OCT was well-preserved in Case 1; therefore, such patients may not complain of any symptoms even though the OCT showed thinning retina and/or EZ disruption.

In addition, the presence of intranuclear inclusions in the inner nuclear layer suggests dysfunction of the cells in the inner nuclear layer, which would also affect the amplitude of ERGs. Moreover, the peripapillary chorioretinal atrophy might be caused by retinal pigment epithelium cell dysfunction associated with the intranuclear inclusions. This pathology may bring reduced thickness of the retina in OCT and abnormal findings in the ERGs.

The frequency of intranuclear inclusions in retinal cells in Case 1 were about 10–25%, which is similar to a previous NIID report on the frequency in the cerebral cortex [33]. However, in Case 2 with an almost normal fundus image with lighter thinning of the retina in OCT than Case1, the frequency of intranuclear inclusions in retinal cells was about 2–10%, which was less than in Case 1. This indicates that the frequency of intranuclear inclusion may correlate with the degree of retinal thinning in OCT. Furthermore, the frequency of intranuclear inclusions in neurons and astrocytes of the frontal lobe in Case 1 was found to be higher than Case 2. This indicates that there is a correlation between retinal thickness in OCT and the severity of retinal pathology, and may be related to the severity of NIID pathology in the cerebrum. Retinal thickness may provide clues to assess the severity of NIID pathology.

On retinal degeneration, we had thought over the co-occurrence of NIID and other distinct retinal dystrophies. However, genetic analysis by WES did not identify any causative gene variants of other retinal diseases in Cases 3 and 4.

This result indicated that the expansion of the GGC repeat of the *NOTCH2NLC* could cause severe retinal degeneration with almost non-recordable ERG and could be clinically diagnosed as retinitis pigmentosa. One study reported that there was no significant correlation between the lengths of the GGC repeats and the age of onset or severity of the disease [37]. However, the numbers of GGC repeats in Cases 3 and 4 were higher than in asymptomatic Cases 1 and 2. Furthermore, Case 2, which had the lowest number of GGC repeats and the mildest fundus findings, had a lower frequency of intranuclear inclusions in retinal cells than Case 1. Although it is suggested that there may be a correlation between the severity of retinal pathology and the number of *NOTCH2NLC* GGC repeats, further studies in more NIID cases are necessary.

Intranuclear inclusions diffusely existed in the entire retina without preference of cell types. This condition may lead to a variety of retinal clinical symptoms and findings including retinal pigment epithelium atrophy, photoreceptor death, bipolar cell dysfunction, or optic atrophy. Additionally, the way of retinal degeneration may differ from those of conventional dystrophies.

Focusing on Cases 3 and 4 with severe retinal degeneration, unlike typical RP, there was a greater propensity for macular involvement. In some previous studies, NIID-related retinopathy showed a peculiar pattern of chorioretinal atrophy which started from the peripapillary region and extended to the macula or the temporal arcade [10, 11, 24]. This unique pattern of retinal impairment was also observed in our Cases 3 and 4. The retinal degeneration in Cases 3 and 4 may be caused not only by photoreceptor degeneration but also by degeneration of the retinal pigment epithelium. In fact, FAF in Case 3 showed widespread hypofluorescence, indicating widespread atrophy of the retinal pigment epithelium. Since retinal pigment epithelium is a single layer of cells, retinal pigment epithelium damage may directly cause photoreceptor degeneration.

SCA7, SCA1, SCA3 and Huntington's disease, polyglutamine diseases caused by expanded CAG repeats, have been reported to cause clinical and experimental retinal degeneration [1, 21, 26, 38]. SCA7 is particularly important in that retinal degeneration has been observed histopathologically in real patients. Retinal findings in our cases shared the similarities with those of SCA7. SCA7 cases showed atrophic macula and intranuclear inclusion in retinal cells [21], and these findings were also observed in our NIID cases. But, migration of melanin pigment toward the atrophic retina in SCA7 was not observed in our NIID cases. Furthermore, in SCA7, intranuclear inclusions are found in the inferior olive, retina, and cerebral cortex, but not in Purkinje cells, which are severely damaged in SCA7. But about NIID, the intranuclear inclusion is widely observed, but the site of neuronal loss varies from case to case [36].

SCA7 and NIID are partially similar in that the distribution of intranuclear inclusions and the site of neuronal loss do not necessarily coincide. This phenomenon may indicate that the expanded repeats sequence brings toxic function, but the intranuclear inclusions visible by light microscopy are not themselves toxic. The molecular mechanisms leading to the discrepancy between intranuclear inclusion distribution and neuronal loss may be similar between SCA7 and NIID. Focusing on this point, it is necessary to elucidate the molecular mechanism of cytotoxicity in these diseases.

Several studies have reported ophthalmologic findings of patients with NIID, ranging from reduction of the ERG signals in the absence of subjective symptoms to severe retinal degeneration resulting in legal blindness [3, 9, 18, 24, 25, 39, 40]. And two case series reported ophthalmologic findings in genetically confirmed NIID patients. In these reports, reduction of amplitude in dark adapted ERG and chorioretinal atrophy in the peripapillary region were described in the examined eyes in spite of the wide range of the severity of retinal degeneration [24]. Our results were similar to the previous case series and the reported two features were also found in our present study.

NIID has been reported to present a very wide distribution of intranuclear inclusions in cells of the central nervous system, peripheral nervous system, and non-neurological tissue. Yet, the distribution of intranuclear inclusions and cell loss did not correlate well. Moreover, neurological manifestations of NIID did not correlate with the distribution of intranuclear inclusions, and oftentimes, intranuclear inclusions were observed in more extensive sites compared to what was expected from clinical manifestations [19, 23, 29, 36].

Because the retina and optic nerve are part of the nervous system, there may be an imbalance between the distribution of intranuclear inclusions in ophthalmologic tissues and ocular symptoms. Additionally, electrophysiological examinations showed delayed conduction velocity and reduced amplitudes in NIID cases [2, 33]. Such nerve conduction abnormalities could also occur in the optic nerve, as intranuclear inclusions are also found in the glial cells of the optic nerve, and may influence the ophthalmologic symptoms. Furthermore, intranuclear inclusions also appear in the optic radiation and the visual cortex of the cerebrum, and it is thought that abnormalities in various parts of the visual pathway including the retina and optic nerve may lead to ophthalmologic symptoms of NIID.

Case 1 presented miosis, and autopsy findings showed almost normal findings of the Edinger-Westphal nucleus that supplies parasympathetic fibres to the eye and constricts the pupil, but many intranuclear inclusions and neuronal loss were observed in the sympathetic ganglia. This pathological imbalance of the damaged sympathetic ganglion and almost intact Edinger-Westphal nucleus may bring parasympathetic nerve dominancy about the pupil diameter, and may be resulting in miosis.

This study has some limitations. One is the incomplete examinations in the asymptomatic patients. We have experienced patients with NIID who were ophthalmologically asymptomatic or who had minimal fundus findings. Although detailed analysis of the retina is required by visual field tests and wide-angle image analysis, most of such patients did not want further ocular examinations. A second limitation is the small number of patients which was probably because that NIID is not yet widely recognized as a disease in the field of ophthalmology. Our cases included sporadic elderly patients, but it is also necessary to confirm whether there are changes in the retina in family cases and/or in younger cases. Third, this study was a cross-sectional study, and it is possible that even patients with no subjective visual symptoms may develop impairment of their visual function from a progression of NIID. Long-term studies of the retinal morphology and function are essential even for NIID patients without visual symptoms.

In conclusion, we confirmed the diffusely scattered distribution of intranuclear inclusions in the retina, gliosis in retina ant optic nerve, and the diversity of the retinal abnormalities in patients with NIID with *NOTCH2NLC* GGC expansion. These results suggested a unique pathology of retinal degeneration in NIID and heterogeneity of ocular manifestations in NIID related to the variety of affected cell types. Autopsy reports of retinal histology in NIID patients are rare. The pathogenicity of NIID is probably different from conventional hereditary retinal diseases. Our findings indicate that visual dysfunction could be the first sign of NIID. We should consider NIID as one of the causes of retinal dystrophy, especially showing an atypical progression of retinal degeneration, and investigate the GGC repeat expansion in *NOTCH2NLC*.

## Supplementary Information

**Additional file 1:** The summary of SNV in RetNet genes.

## Data Availability

The datasets generated and/or analysed during the current study are not publicly available due to protection of personal information but are available from the corresponding author on reasonable request.

## Abbreviations

NIID: Neuronal intranuclear inclusion disease
DWI: Diffusion weighted image
MRI: Magnetic resonance imaging
RP: Retinitis pigmentosa
INL: Inner nuclear layer
BCVA: Best-corrected visual acuity
ERG: Electroretinogram
SD-OCT: Spectral domain optical coherence tomographic
FAF: Fundus autofluorescence
RPE: Retinal pigment epithelium
GCL: Ganglion cell layer
ONL: Outer nuclear layer
GFAP: Glial fibrillary acidic protein
PCR: Polymerase chain reaction
WES: Whole exome sequencing
IGV: Integrative Genomics Viewer
MMSE: Mini-mental state examination
FAB: Frontal assessment battery
D.A.: Dark-adapted
L.A.: Light-adapted
FXTAS: Fragile X-associated tremor/ataxia syndrome
OPDM: Oculopharyngodistal myopathy

## References

[1] Abe T, Abe K, Aoki M, Itoyama Y, Tamai M (1997). Ocular changes in patients with spinocerebellar degeneration and repeated trinucleotide expansion of spinocerebellar ataxia type 1 gene. Arch Ophthalmol.

[2] Araki K, Sone J, Fujioka Y, Masuda M, Ohdake R, Tanaka Y, Nakamura T, Watanabe H, Sobue G (2016). Memory loss and frontal cognitive dysfunction in a patient with adult-onset neuronal intranuclear inclusion disease. Intern Med.

[3] Arrindell EL, Trobe JD, Sieving PA, Barnett JL (1991). Pupillary and electroretinographic abnormalities in a family with neuronal intranuclear hyaline inclusion disease. Arch Ophthalmol.

[4] Boivin M, Deng J, Pfister V, Grandgirard E, Oulad-Abdelghani M, Morlet B, Ruffenach F, Negroni L, Koebel P, Jacob H (2021). Translation of GGC repeat expansions into a toxic polyglycine protein in NIID defines a novel class of human genetic disorders: The polyG diseases. Neuron.

[5] Deng J, Yu J, Li P, Luan X, Cao L, Zhao J, Yu M, Zhang W, Lv H, Xie Z (2020). Expansion of GGC Repeat in GIPC1 is associated with oculopharyngodistal myopathy. Am J Hum Genet.

[6] Escartin C, Galea E, Lakatos A, O'Callaghan JP, Petzold GC, Serrano-Pozo A, Steinhauser C, Volterra A, Carmignoto G, Agarwal A (2021). Reactive astrocyte nomenclature, definitions, and future directions. Nat Neurosci.

[7] Hagerman P (2013). Fragile X-associated tremor/ataxia syndrome (FXTAS): pathology and mechanisms. Acta Neuropathol.

[8] Haltia M, Somer H, Palo J, Johnson WG (1984). Neuronal intranuclear inclusion disease in identical twins. Ann Neurol.

[9] Haltia M, Tarkkanen A, Somer H, Palo J, Karli H (1986). Neuronal intranuclear inclusion disease. Clinical ophthalmological features and ophthalmic pathology. Acta Ophthalmol (Copenh).

[10] Hayashi T, Katagiri S, Mizobuchi K, Yoshitake K, Kameya S, Matsuura T, Iwata T, Nakano T (2020). Heterozygous GGC repeat expansion of NOTCH2NLC in a patient with neuronal intranuclear inclusion disease and progressive retinal dystrophy. Ophthalmic Genet.

[11] Hsia Y, Cheng CY, Tang SC, Lin CW (2021). Peculiar pattern of retinopathy in adult-onset neuronal intranuclear inclusion disease. J Formos Med Assoc.

[12] Ishiura H, Shibata S, Yoshimura J, Suzuki Y, Qu W, Doi K, Almansour MA, Kikuchi JK, Taira M, Mitsui J (2019). Noncoding CGG repeat expansions in neuronal intranuclear inclusion disease, oculopharyngodistal myopathy and an overlapping disease. Nat Genet.

[13] Karczewski KJ, Francioli LC, Tiao G, Cummings BB, Alföldi J, Wang Q, Collins RL, Laricchia KM, Ganna A, Birnbaum DP (2020). The mutational constraint spectrum quantified from variation in 141,456 humans. Nature.

[14] Koob MD, Benzow KA, Bird TD, Day JW, Moseley ML, Ranum LP (1998). Rapid cloning of expanded trinucleotide repeat sequences from genomic DNA. Nat Genet.

[15] Koyanagi Y, Akiyama M, Nishiguchi KM, Momozawa Y, Kamatani Y, Takata S, Inai C, Iwasaki Y, Kumano M, Murakami Y (2019). Genetic characteristics of retinitis pigmentosa in 1204 Japanese patients. J Med Genet.

[16] Landrum MJ, Lee JM, Benson M, Brown GR, Chao C, Chitipiralla S, Gu B, Hart J, Hoffman D, Jang W (2018). ClinVar: improving access to variant interpretations and supporting evidence. Nucleic Acids Res.

[17] Lindenberg R, Rubinstein LJ, Herman MM, Haydon GB (1968). A light and electron microscopy study of an unusual widespread nuclear inclusion body disease. A possible residuum of an old herpesvirus infection. Acta Neuropathol (Berl).

[18] Liu C, Luan X, Liu X, Wang X, Cai X, Li T, Cao L, Long D (2021). Characteristics of ocular findings of patients with neuronal intranuclear inclusion disease. Neurol Sci.

[19] Liu Y, Mimuro M, Yoshida M, Hashizume Y, Niwa H, Miyao S, Ujihira N, Akatsu H (2008). Inclusion-positive cell types in adult-onset intranuclear inclusion body disease: implications for clinical diagnosis. Acta Neuropathol.

[20] McCulloch DL, Marmor MF, Brigell MG, Hamilton R, Holder GE, Tzekov R, Bach M (2015). ISCEV Standard for full-field clinical electroretinography (2015 update). Doc Ophthalmol.

[21] Michalik A, Martin JJ, Van Broeckhoven C (2004). Spinocerebellar ataxia type 7 associated with pigmentary retinal dystrophy. Eur J Hum Genet.

[22] Michaud J, Gilbert JJ (1981). Multiple system atrophy with neuronal intranuclear hyaline inclusions. Report of a new case with light and electron microscopic studies. Acta Neuropathol (Berl).

[23] Munoz-Garcia D, Ludwin SK (1986). Adult-onset neuronal intranuclear hyaline inclusion disease. Neurology.

[24] Nakamura N, Tsunoda K, Mitsutake A, Shibata S, Mano T, Nagashima Y, Ishiura H, Iwata A, Toda T, Tsuji S (2020). Clinical characteristics of neuronal intranuclear inclusion disease-related retinopathy with CGG repeat expansions in the NOTCH2NLC gene. Invest Ophthalmol Vis Sci.

[25] Omoto S, Hayashi T, Matsuno H, Higa H, Kameya S, Sengoku R, Takahashi-Fujigasaki J, Murayama S, Iguchi Y (2018). Neuronal intranuclear hyaline inclusion disease presenting with childhood-onset night blindness associated with progressive retinal dystrophy. J Neurol Sci.

[26] Petrasch-Parwez E, Saft C, Schlichting A, Andrich J, Napirei M, Arning L, Wieczorek S, Dermietzel R, Epplen JT (2005). Is the retina affected in Huntington disease?. Acta Neuropathol.

[27] RetNet RetNet https://sph.uth.edu/retnet/. Accessed June 22, 2021

[28] Saito R, Shimizu H, Miura T, Hara N, Mezaki N, Higuchi Y, Miyashita A, Kawachi I, Sanpei K, Honma Y (2020). Oculopharyngodistal myopathy with coexisting histology of systemic neuronal intranuclear inclusion disease: clinicopathologic features of an autopsied patient harboring CGG repeat expansions in LRP12. Acta Neuropathol Commun.

[29] Sone J, Hishikawa N, Koike H, Hattori N, Hirayama M, Nagamatsu M, Yamamoto M, Tanaka F, Yoshida M, Hashizume Y (2005). Neuronal intranuclear hyaline inclusion disease showing motor-sensory and autonomic neuropathy. Neurology.

[30] Sone J, Kitagawa N, Sugawara E, Iguchi M, Nakamura R, Koike H, Iwasaki Y, Yoshida M, Takahashi T, Chiba S (2014). Neuronal intranuclear inclusion disease cases with leukoencephalopathy diagnosed via skin biopsy. J Neurol Neurosurg Psychiatry.

[31] Sone J, Mitsuhashi S, Fujita A, Mizuguchi T, Hamanaka K, Mori K, Koike H, Hashiguchi A, Takashima H, Sugiyama H (2019). Long-read sequencing identifies GGC repeat expansions in NOTCH2NLC associated with neuronal intranuclear inclusion disease. Nat Genet.

[32] Sone J, Mitsuhashi S, Fujita A, Mizuguchi T, Mori K, Koike H, Hashiguchi A, Takashima H, Sugiyama H, Kohno Y (2019). Long-read sequencing identifies GGC repeat expansion in human-specific NOTCH2NLC associated with neuronal intranuclear inclusion disease. Biorxiv.

[33] Sone J, Mori K, Inagaki T, Katsumata R, Takagi S, Yokoi S, Araki K, Kato T, Nakamura T, Koike H (2016). Clinicopathological features of adult-onset neuronal intranuclear inclusion disease. Brain.

[34] Sone J, Tanaka F, Koike H, Inukai A, Katsuno M, Yoshida M, Watanabe H, Sobue G (2011). Skin biopsy is useful for the antemortem diagnosis of neuronal intranuclear inclusion disease. Neurology.

[35] Tadaka S, Hishinuma E, Komaki S, Motoike IN, Kawashima J, Saigusa D, Inoue J, Takayama J, Okamura Y, Aoki Y (2021). jMorp updates in 2020: large enhancement of multi-omics data resources on the general Japanese population. Nucleic Acids Res.

[36] Takahashi-Fujigasaki J (2003). Neuronal intranuclear hyaline inclusion disease. Neuropathology.

[37] Tian Y, Wang JL, Huang W, Zeng S, Jiao B, Liu Z, Chen Z, Li Y, Wang Y, Min HX (2019). Expansion of human-specific GGC repeat in neuronal intranuclear inclusion disease-related disorders. Am J Hum Genet.

[38] Toulis V, Casaroli-Marano R, Camos-Carreras A, Figueras-Roca M, Sanchez-Dalmau B, Munoz E, Ashraf NS, Ferreira AF, Khan N, Marfany G et al (2022) Altered retinal structure and function in Spinocerebellar ataxia type 3. Neurobiol Dis 170. 10.1016/j.nbd.2022.105774 10.1016/j.nbd.2022.105774 35605759

[39] Yamada W, Takekoshi A, Ishida K, Mochizuki K, Sone J, Sobue G, Hayashi Y, Inuzuka T, Miyake Y (2017). Case of adult-onset neuronal intranuclear hyaline inclusion disease with negative electroretinogram. Doc Ophthalmol.

[40] Yamaguchi N, Mano T, Ohtomo R, Ishiura H, Almansour MA, Mori H, Kanda J, Shirota Y, Taira K, Morikawa T (2018). An autopsy case of familial neuronal intranuclear inclusion disease with dementia and neuropathy. Intern Med.

